# Tolerability of Eribulin and correlation between polymorphisms and neuropathy in an unselected population of female patients with metastatic breast cancer: results of the multicenter, single arm, phase IV PAINTER study

**DOI:** 10.1186/s13058-022-01560-w

**Published:** 2022-10-28

**Authors:** Nicla La Verde, Giovanna Damia, Ornella Garrone, Daniele Santini, Alessandra Fabi, Mariangela Ciccarese, Daniele Giulio Generali, Martina Nunzi, Elena Poletto, Elisa Ferraris, Elisabetta Cretella, Giuseppa Scandurra, Icro Meattini, Alessandro Stefano Bertolini, Luigi Cavanna, Elena Collovà, Emanuela Romagnoli, Eliana Rulli, Lorenzo Legramandi, Federica Guffanti, Annalisa Bramati, Anna Moretti, Alessandra Cassano, Patrizia Vici, Valter Torri, Gabriella Farina, PAINTER investigators

**Affiliations:** 1 Department of Oncology, Luigi Sacco Hospital, ASST Fatebenefratelli Sacco, Milan, Italy; 2 Laboratory of Molecular Pharmacology, Istituto Di Ricerche Farmacologiche Mario Negri IRCCS, Milan, Italy; 3 Medical Oncology, Fondazione IRCCS Ca’ Granda Ospedale Maggiore Policlinico, Milan, Italy; 4 Oncologia Medica Università Campus Biomedico, Rome, Italy; 5 UOC Oncologia Universitaria, Sapienza University of Rome Polo Pontino, Latina, Italy; 6 Fondazione Policlinico Universitario A. Gemelli, IRCCS, Rome, Italy; 7 Dept of Oncology Vito Fazzi Hospital, Lecce, Italy; 8 U.O. Multidisciplinare Di Patologia Mammaria E Ricerca, Traslazionale Azienda Socio-Sanitaria Territoriale Di Cremona, Cremona, Italy; 9 Dept. of Oncology Medical and Translational Oncology Unit, Azienda Ospedaliera S.Maria, Terni, Italy; 10 ASUFC Presidio Ospedaliero Universitario S.M. Misericordia, Udine, Italy; 11 Division of Medical Oncology, IRCCS San Matteo University Hospital Foundation, Pavia, Italy; 12 Oncologia Medica Ospedale Di Bolzano, AS Alto Adige, Bolzano, Italy; 13 Medical Oncology Unit, Cannizzaro Hospital, Catania, Italy; 14 Radiation Oncology Unit, Oncology Department, Azienda Ospedaliera Universitaria Careggi, Florence, Italy; 15 SOC Oncologia Medica, ASST Della Valtellina E Alto Lario, Sondrio, Italy; 16 Oncology Haematology Department, ASL Piacenza, Piacenza, Italy; 17 ASST Ovest Milanese, Ospedale Di Legnano, Legnano, MI Italy; 18 UOC Oncologia, Ospedale Macerata, Macerata, Italy; 19 Laboratory of Methodology for Clinical Research, Istituto Di Ricerche Farmacologiche Mario Negri IRCCS, Milan, Italy; 20 Department of Oncology, Fatebenefratelli Hospital, ASST Fatebenefratelli-Sacco, Piazza Principessa Clotilde 3, 20121 Milan, Italy; 21 Comprehensive Cancer Center, Fondazione Policlinico Universitario Agostino Gemelli, IRCCS, Rome, Italy; 22 Phase IV Clinical Studies Unit, IRCCS Regina Elena National Cancer Institute, Rome, Italy; 23 Department of Oncology, Istituto Di Ricerche Farmacologiche Mario Negri IRCCS, Milan, Italy

**Keywords:** Breast cancer, Eribulin, Neurotoxicity, Polymorphisms, Peripheral neuropathy

## Abstract

**Background:**

Metastatic breast cancer (MBC) is an incurable disease and its treatment focuses on prolonging patients’ (pts) overall survival (OS) and improving their quality of life. Eribulin is a microtubule inhibitor that increases OS in pre-treated MBC pts. The most common adverse events (AEs) are asthenia, neutropenia and peripheral neuropathy (PN).

**Methods:**

PAINTER is a single arm, phase IV study, aimed at evaluating the tolerability of eribulin in MBC pts. Secondary objectives were the description of treatment efficacy and safety, the assessment of the incidence and severity of PN and its association with genetic polymorphisms. Genomic DNA was isolated from blood samples and 15 Single Nucleotide Polymorphisms (SNPs) were genotyped by Taqman specific assays. The association between PN and SNPs were evaluated by Fisher exact test.

**Results:**

Starting from May 2014 until June 2018 180 pts were enrolled in this study by 20 Italian centers. 170 of these pts could be evaluated for efficacy and toxicity and 159 for polymorphisms analysis. The median age of pts was 60 years old and the biological subtypes were luminal type (64.7%), Her2 positive (18.3%) and triple negative (17%). Pts were pretreated with a median of 5 lines for MBC. The median follow up of this study was 15.4 months with a median number of 4.5 cycles administered (minimum–maximum 1–23). The median overall survival was 12 months. 48.8% of pts experienced a dose reduction, mainly for neutropenia (23.9%) and liver toxicity (12%). 65 pts (38.2%) reported at least one severe toxicity. Neutropenia and neurotoxicity were the most frequent severe AEs (15.3% and 14.7%, respectively); other reported toxicities were osteo-muscular, abdominal or tumor site pain (19.4%), liver toxicity (6.6%), pulmonary toxicity (6.5%) and dermatological toxicity (3.6%). Among the 15 evaluated SNPs, an association with PN was found for rs2233335 and rs7214723.

**Conclusions:**

Eribulin is a well-tolerated treatment option in MBC. Schedule and dosage modifications were common, but toxicity rarely led to treatment discontinuation. SNPs rs2233335 (G/T and T/T) in the *NDRG1* gene and rs7214723 (CC and CT) in the *CAMKK1* gene were associated with PN. These findings, if validated, could allow a tailored treatment with eribulin in cancer patients.

*Trial registration*: ClinicalTrials.gov ID: NCT02864030.

**Supplementary information:**

The online version contains supplementary material available at 10.1186/s13058-022-01560-w.

## Background

Metastatic breast cancer is an incurable disease, with a median survival ranging from 24 to 48 months, which varies according to biological characteristics, metastatic sites, patients’ age, etc. [1, 2]. Treatments are tailored based patients and tumor characteristics, and the use of new drugs, such as CDK4/6 inhibitors or monoclonal antibodies significantly improved disease control [3, 4]. Despite the development of new agents, single-agent chemotherapy remains an important backbone in the metastatic setting. Capecitabine, vinorelbine and eribulin are the preferred options in patients who have already been treated with anthracyclines and taxanes [2].

Eribulin is a synthetic analog of the natural product halichondrin B. Its cytotoxic effects are mainly due to its ability to interfere with microtubule dynamics by causing the blockage of mitotic spindle formation, mitotic arrest and subsequent cell death by apoptosis [5]. Differently from other microtubule interfering agents, eribulin inhibits the growth phase of the microtubules without any inhibition of the shortening phase [6–8]. However, many other non-mitotic effects of eribulin on tumor biology have been described, including tumor vascular remodeling, which leads to better tumor perfusion and reduced hypoxia, and interference with epithelial mesenchymal transition which reduces the ability of tumor cells to migrate and invade, both in vitro and in vivo. These latter effects of eribulin seem to be relevant in justifying the clinical activity observed with this drug [9].

Eribulin has been approved by the Food and Drug Administration in 2010 and in Italy in 2012 for the treatment of patients with locally advanced or metastatic breast cancer which has progressed after at least one chemotherapy regimen, including a combination of anthracyclines and taxanes in both the adjuvant and metastatic setting.

The approval of this drug in breast cancer was based on the results of two randomized phase III trials [10, 11]. The first trial EMBRACE [10] enrolled 762 women with locally recurrent or MBC randomly assigned (2:1) to eribulin or a treatment of physician’s choice (TPC). Overall survival was significantly improved in the experimental arm (median 13.1 months, 95% CI 11.8–14.3) compared to TPC arm (10.6 months, 9.3–12.5; hazard ratio 0.81, 95% CI 0.66–0.99; p = 0.041). The second trial, the 301 study [11], evaluated the efficacy and safety of eribulin as first, second or third line monotherapy versus capecitabine in 1102 patients with locally advanced breast cancer or MBC who had been previously treated with anthracyclines and taxanes. The median OS was 15.9 months for eribulin versus 14.5 months for capecitabine (HR 0.879; 95% CI: 0.770–1.003; p = 0.056), and the study failed to demonstrate the superiority of eribulin. A pooled analysis suggested a major benefit in the subgroup of women with HER2 negative disease [12]. Results from phase III trials suggested that eribulin was well tolerated and the most common adverse effects (AEs) were neutropenia, fatigue and peripheral neuropathy. Specifically, this latter AE occurred in 5% of patients enrolled in the EMBRACE study and in 13% of patients enrolled in the 301 study. In a recent meta-analysis, the incidence of all-grade and high-grade peripheral neuropathy after treatment with Eribulin was 27.5% (95% CI: 23.3–32.4%) and 4.7% (95% CI: 3.6–6.2%), respectively [13]. Even though the precise mechanism behind the neurotoxicity caused by microtubule interfering agents (taxanes, vinca alkaloids and eribulin) has not been fully defined yet, preclinical and histological studies suggest that its pathogenesis is mainly a consequence of the interruption of the axonal transport within the neuron, which relies on intact microtubule structures [14, 15]. The prevalence of severe neuropathy is extremely variable, suggesting that individual characteristics might affect susceptibility. For this reason, the study of gene polymorphisms could help in identifying patients at a higher risk of developing neurotoxicity, as has already been demonstrated for taxanes [14, 16].

In this paper we report the results of the PAINTER study which aimed to evaluate the toxicity and quality of life (QoL) in unselected Italian patients with MBC treated with eribulin. The correlation between neurotoxicity and SNPs was also explored.

## Methods

### Study design and participants

The PAINTER study is a single arm, phase IV, multicentre study with the primary objective of surveying the tolerability profile of eribulin in an unselected population of patients with MBC (real life setting). The secondary objectives were the study of the relationship between specific genetic polymorphisms and the incidence and severity of peripheral neuropathy and the description of treatment efficacy in terms of duration of treatment and patient survival.

The study population included patients diagnosed with MBC treated with eribulin in accordance with the guidelines of the Italian regulatory Authority. All patients received an intravenous infusion of 1.23 mg/m^2^ eribulin on days 1 and 8 on a 21-day cycle. Treatments cycles were repeated until disease progression, unacceptable toxicity, patient refusal or medical decision. The physician could choose any further line of treatment after disease progression. Patients were monitored in order to identify any AEs during treatment with eribulin and up to 30 days after its discontinuation. Follow-up visits for survival assessment were performed every 4 months.

QoL was evaluated on day 1 of every cycle, and 30 days after the discontinuation of treatment using EORTC QLQ-C30 and QLQ-BR23 questionnaires.

The study complied with the Declaration of Helsinki. It was performed according to Good Clinical Practice guidelines and was approved by the local ethic committees in all the participating centers. All patients provided written informed consent. The study protocol is registered with ClinicalTrials.gov NCT02864030.

### Endpoints

The study endpoints were:

- incidence, time of onset, severity and duration of all AEs experienced during treatment with eribulin, including the most common toxicities reported in previous studies (asthenia/fatigue, neutropenia, peripheral neurotoxicity, constipation alopecia, nausea) as well as other possible unexpected toxicities. The primary analysis was based on severe AEs (SAEs) defined as grade 3 or 4 AEs, except for neuropathy and alopecia for which a grade 2 was considered severe;
- assessment of dose intensity and dose schedule maintenance as indirect index of tolerability;
- Duration of treatment (DOT) and Overall Survival (OS).
- evaluation of QoL during treatment, using validated questionnaires;
- the association between a set of selected SNPs and the occurrence of any grade peripheral neuropathy. Specifically, 15 SNPs located in genes involved in microtubule dynamics or identified in genome wide association studies were analyzed.

### Sample size

The planned sample size was 200 patients, which was defined taking into account temporal and logistic constraints.

For the purpose of the evaluation, the severe toxicity (Grade 3 or 4) was chosen as a safety endpoint of primary interest. A sample size of 200 patients, considering a toxicity rate between 20 and 40%, produces a 95% confidence interval (95% CI) with an amplitude of at most 14%, deemed sufficiently precise to draw valid conclusions on the event rate. The estimation of the 95% CI was based on the Clopper-Pearson methodology [17]. Moreover, a sample size of 200 patients was deemed adequate for the statistical analysis of the relationship between the primary endpoint and not more than 10 factors [18]. In addition it enabled us to study the relationship between 10–15 polymorphisms, with a known prevalence > 15%, and the risk of neuropathy. Assuming a risk of neuropathy of 30%, and a clinically relevant association in terms of odds ratio (OR) of 3, the study had 80% power to detect a statistically significant association at 2.5% one side level for each assessment.

### Statistical methods

Eribulin safety was analyzed on the “safety patients set”, which included patients who had received at least one dose of treatment. For those patients included in the safety set whose blood samples were available, the evaluation of polymorphisms was also performed (“molecular analysis patients set”). AEs were described using the maximum grade observed during the treatment. The SAEs were described by means of absolute and relative frequencies and associated 95% CI estimated by means of exact binomial methods. The occurrence of neuropathy was described using the cumulative incidences of any grade and severe grade (equal or greater than grade 2) by means of Kaplan–Meier methods.

The relationship between polymorphisms and risk of neuropathy was described by contingency tables and their association was assessed by χ2 test for trend and a Fisher exact test in order to detect one of two different pathways of association, linear or dominant model. For those polymorphisms that resulted associated to neuropathy with the previous tests, the association with severe (G2-3–4) neuropathy was assessed by a univariable logistic model. No multivariable model was planned because of the low number of severe neuropathy events.

DOT was calculated as the time from the start of eribulin treatment to its discontinuation. OS was calculated as the time from the beginning of treatment start to the date of death from any cause. Patients who were alive at the end of the study were censored at the last date they were known to be alive. DOT and OS were described using Kaplan–Meier curves. The cumulative incidences for the two competitive events of interest (toxicity and progression) were calculated for DOT analysis according to the Fine and Grey’s method.

QoL scores at 3 months and at the end of treatment were compared with baseline scores for each patient and were evaluated separately for each questionnaire scale. A paired T-test was used to analyze changes. A p value < 0.05 was considered statistically significant. The analysis was exploratory in nature and for this reason no adjustment for multiple assessment was planned. All analyses were performed using SAS Version 9.4.

### SNPs analysis

For the determination of polymorphisms, blood was collected in a Vacutainer containing EDTA any time during the participant’s first two treatment cycles and stored at -20° Celsius until further processing. Genomic DNA was purified from whole blood samples using the Maxwell® RSC Whole Blood DNA kit (Promega, Italy). DNA was amplified using the TaqMan® Genotyping Master Mix (Thermo Fisher Scientific, USA) and analyzed according to manufacturer’s instructions for the presence of selected SNP allele variants by real-time PCR technique (ABI-7900; Applied Biosystems, Italy) using TaqMan SNP Genotyping assays (Thermo Fisher Scientific, USA) specific for each gene of interest. Additional file 1: Table S1 shows the SNPs analyzed, selected based on their reported association with neuropathy induced by anticancer agents*.* Real-time PCR was carried out in 384-wells plates prepared with automatic liquid handling (epMotion 5075; Eppendorf, Italy). Completed PCR plates were analyzed using the TaqMan® Genotyper Software (Thermo Fisher Scientific, USA).

## Results

**Fig. 1:**
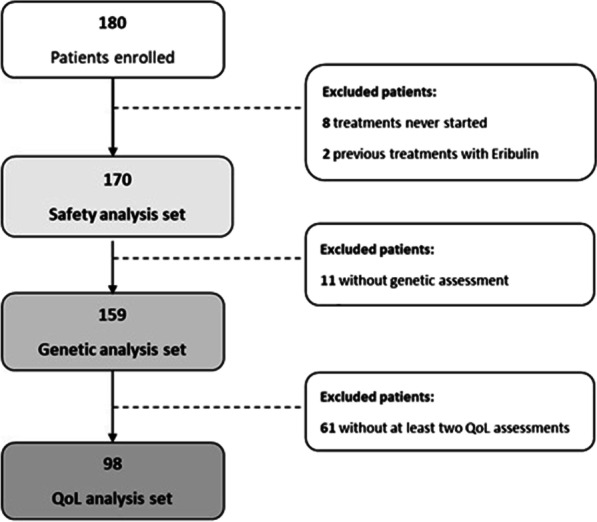
Study flow chart

Starting from May 2014 until June 2018, 180 patients were enrolled in this study by 20 different Italian centers. As depicted in the Flowchart (Fig. 1), 10 patients were excluded from the safety analysis: 2 patients due to major protocol violations (having received eribulin in previous lines of treatment) and 8 patients because they never started treatment with eribulin. Only 159 patients out of 180 were included in the analysis of polymorphisms (molecular analysis set), since the blood samples of 11 patients were not available. Ninety-eight patients (54.4%) were considered for QoL assessment. The median follow-up was 15.4 months.

The main baseline characteristics of patients, according to the analysis sets, are summarized in Table 1. Overall, mean patient age was 59.7, 64% had a Luminal HER-2 negative metastatic breast cancer, ECOG Performance status was 0–1 in 100% of the patients, and the mean number of previous chemotherapy lines for metastatic disease was 5 (from 0 to a maximum of 18). Previous neuropathy was reported in 15.9% and 17% of patients in the safety and molecular analysis sets, respectively.

**Table 1:** Demographic and Baseline Characteristics

	Molecular analysis set N = 159	Safety analysis set N = 170
Age		
Mean (SD)	59.9 (11.9)	59.7 (12.0)
Median (Q1-Q3)	60.0 (51.0–69.0)	60.0 (51.0–69.0)
BMI (Kg/m^2^)		
Mean (SD)	25.2 (4.9)	25.5 (4.9)
Median (Q1-Q3)	24.5 (21.8–28.4)	24.7 (22.0–28.5)
Time from diagnosis (years)		
Mean (SD)	7.8 (5.8)	7.9 (5.8)
Median (Q1-Q3)	6.0 (3.4–11.0)	6.1 (3.5–11.0)
Histology—*n* (%)		
Ductal	120 (85.7)	129 (86.6)
Lobular	20 (14.3)	20 (13.4)
Unknown/missing	19 (11.9)	21 (12.3)
Biological subtype—*n* (%)		
HER2 positive	27 (18.8)	28 (18.3)
Luminal	91 (63.2)	99 (64.7)
Triple Negative	26 (18.1)	26 (17.0)
Missing	15 (9.4)	17 (10)
Metastasis—*n* (%)		
Not visceral	50 (31.4)	53 (31.2)
Visceral	109 (68.6)	117 (68.8)
Number of therapy lines in metastatic setting—*n* (%)		
Mean (SD)	5.0 (3.1)	5.0 (3.0)
Median (Q1-Q3)	5.0 (3.0–7.0)	5.0 (3.0–7.0)
Missing	3 (1.9)	3 (1.7)
Baseline neurotoxicity—*n* (%)		
No	132 (83.0)	143 (84.1)
Yes	27 (17.0)	27 (15.9)

The median number of eribulin cycles administered per patient was 4.5 (first quartile[Q1]- third quartile[Q3] 3.0–7.0) from a minimum of 1 to a maximum of 23 cycles. Half of the patients received 84.7% of the cycles at the full dose but the treatment was modified for 83 patients (48.8%), mostly before the 3^rd^ cycle. The main reasons for treatment discontinuation were disease progression (82.9%), loss to follow up (4.7%), medical decision (4.7%) and toxicity (3.5%).

### Efficacy

**Fig. 2:**
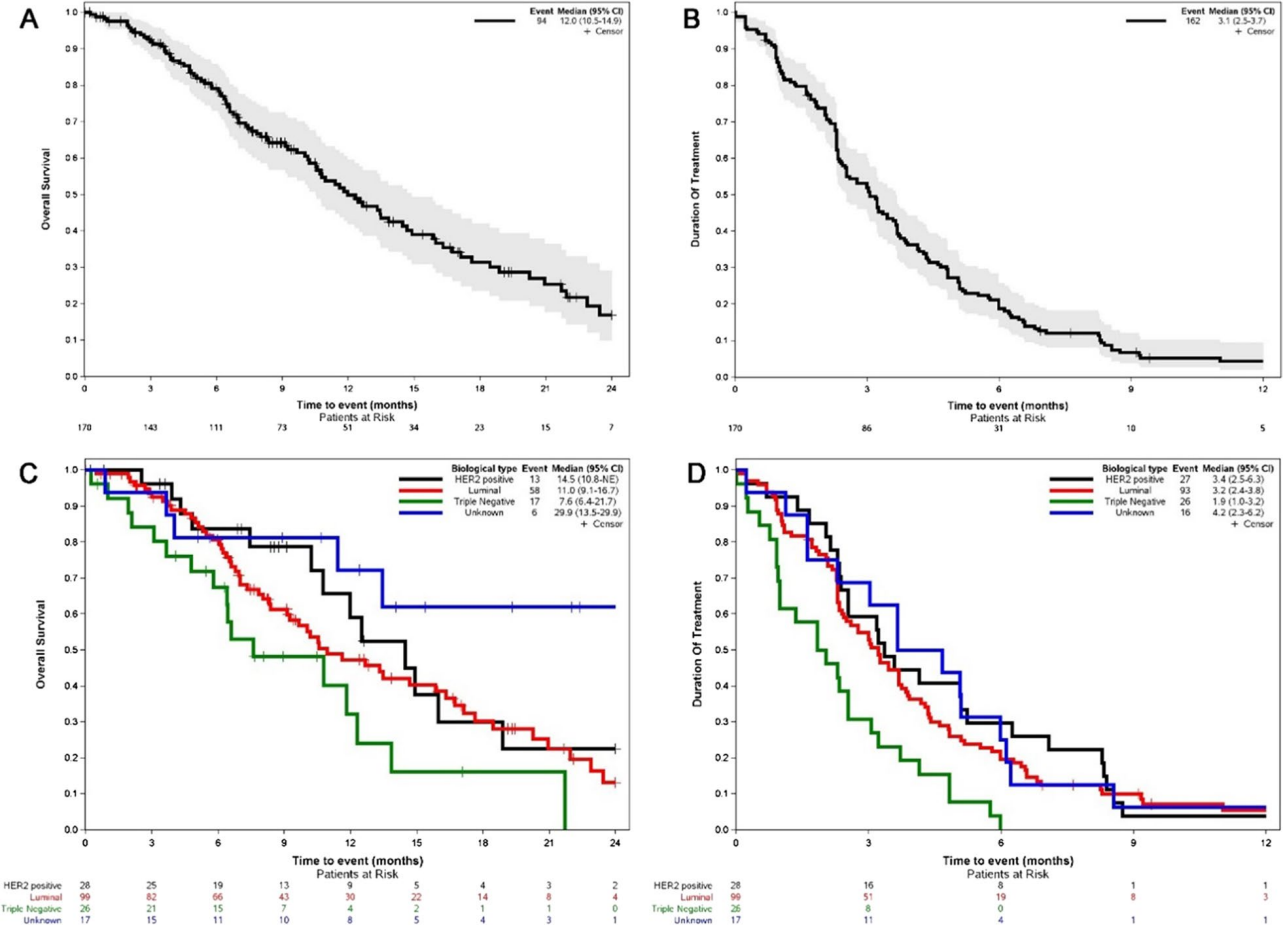
**A** Overall population OS;** B** Overall population DOT; **C** OS based on biological subtypes; **D** DOT based on biological subtypes

At a median follow-up of 15.4 months, 94 (55.3%) patients had died, mainly due to disease progression (96.8%). The median OS was 12 months (Q1-Q3: 6.4–21.7 months) (Fig. 2A). The OS was 79.1% and 49.8% at 6 and 12 months, respectively. At the time point of the statistical analysis, 8 patients were lost to follow-up and therefore considered as censored for the DOT analysis, while 162 (95.3%) had discontinued treatment. The median DOT was 3.1 months (Q1-Q3: 1.8–5.1 months) (Fig. 2B). Out of 182 patients, 145 (85.3%) had discontinued treatment because of inefficacy, while 17 (10%) because of toxicity or patient/medical decision. The cumulative incidence for interruption due to inefficacy was 42.1% (95%CI: 34.5–49.5) and 72.3% (95%CI: 64.7–78.5) at 3 and 6 months, respectively. The cumulative incidence for interruption due toxicity was 5.9% (95%CI: 3.-10.3) and 9.00% (95%CI: 5.3–13.9) at 3 and 6 months, respectively.

#### Toxicity

Table 2 shows the overall incidence of AEs. Overall, 72 (42.4%) patients experienced at least one severe toxicity. Additional file 1: Table S2 reports all the recorded adverse events. The most common toxicities occurred were neutropenia (patients with G3-G5: 15.3%; 95%CI 10.2 – 21.6) and neurotoxicity (patients with G2-G5: 14.7%; 95%CI: 9.75 – 20.9).

**Table 2:** Description of the main adverse events

Toxicity *N* = 170	G0 *n* (%)	G1 *n* (%)	G2 *n* (%)	G3 *n* (%)	G4 *n* (%)	G5 *n* (%)	Severe toxicity (G3 + G4 + G5*) *n* (%) [%95%CI]
Overall adverse events	26 (15.3)	35 (20.6)	58 (34.1)	38 (22.4)	12 (7.1)	1 (0.6)	65 (38.2) [30.9—46.0]
Neurotoxicity	114 (67.1)	31 (18.2)	20 (11.8)	5 (2.9)	0 (0.0)	0 (0.0)	25 (14.7)*[9.75—20.9]
Neutropenia	118 (69.4)	15 (8.8)	11 (6.5)	16 (9.4)	10 (5.9)	0 (0.0)	26 (15.3) [10.2—21.6]
Constipation	145 (85.3)	16 (9.4)	8 (4.7)	1 (0.6)	0 (0.0)	0 (0.0)	1 (0.6) [0.015—3.23]
Alopecia	132 (77.6)	25 (14.7)	13 (7.6)	0 (0.0)	0 (0.0)	0 (0.0)	0 (0.0)
Asthenia	84 (49.4)	38 (22.4)	38 (22.4)	10 (5.9)	0 (0.0)	0 (0.0)	10 (5.9) [2.86—10.6]
Nausea	145 (85.3)	19 (11.2)	6 (3.5)	0 (0.0)	0 (0.0)	0 (0.0)	0 (0.0)

N: number of subjects; G: Grade; *: G2 was considered as severe toxicity for neurotoxicity.

**Fig. 3:**
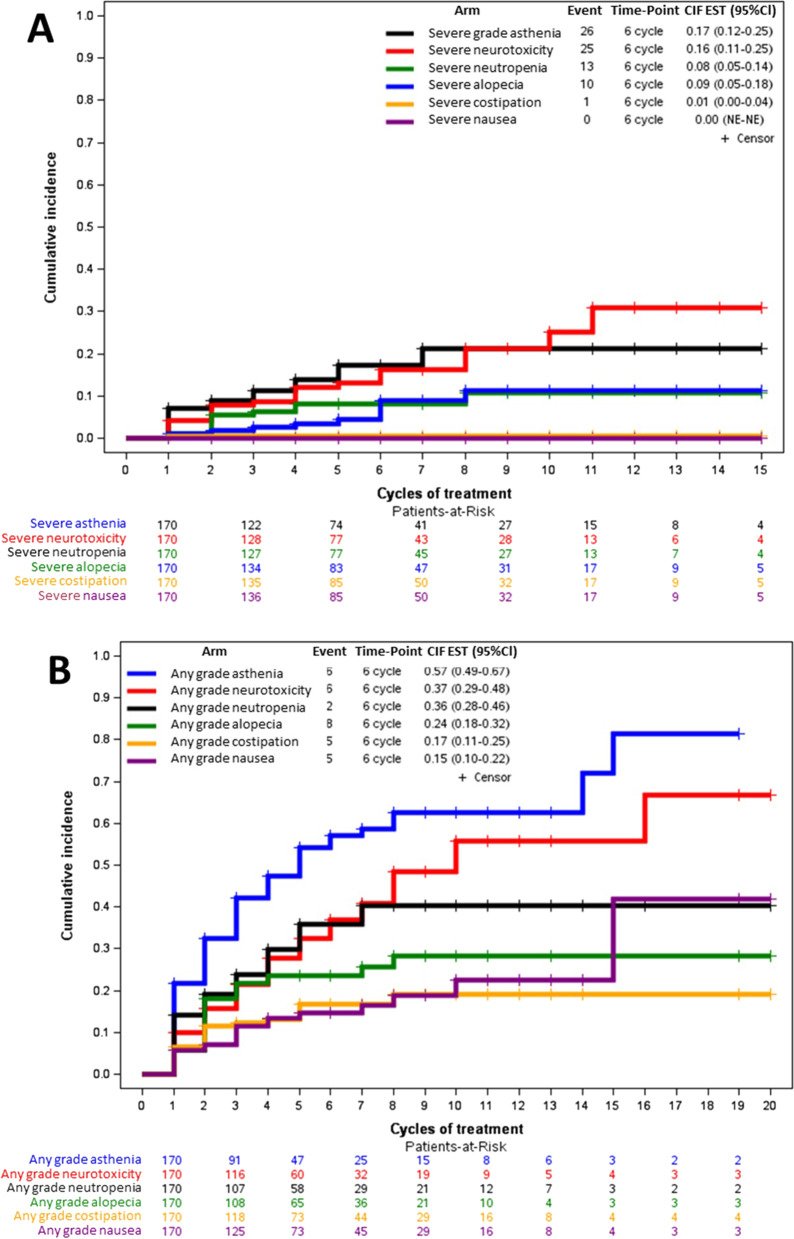
Cumulative incidence of severe toxicities (**A**) and any grade toxicity (**B**)

The cumulative incidence of toxicities is described in Fig. 3 (3A for severe toxicities, 3B for all grade toxicities). The incidence of neurotoxicity in the first 5 cycles was 32.5% and 13.2% referred to any grade and grades > 1, respectively. The incidence of neutropenia occurrence in the first 5 cycles was 35.9% and 17.3% referred to any grade and grades > 2, respectively.

We have explored the association between having a previous neurotoxicity and the occurrence of neurotoxicity during the eribulin treatment. We found that the previous neuropathy is associated to a higher risk of high grade of neuropathy during eribulin (Chi for trend = 13.60 p = 0.0002—Table 3). Despite this association, the previous neuropathy does not affect the number of cycles of eribulin received (previous neuroxicity median cycles:4 Q1-Q3:3–7; no previous neurotoxicity median cycles:5 Q1-Q3:3–8; wilcoxcon test P = 0.4187, see Additional file 1: Table S6). No correlation was found between neurotoxicity and OS.

**Table 3:** Neurotoxicity based on previous patients’ neuropathy

Previous neurotoxicity	Neurotoxicity during eribulin treatment				
	*G0*	*G1*	*G2*	*G3*	*Total*
	*n (%)*	*n (%)*	*n (%)*	*n (%)*	
No	105 (73.4)	19 (13.3)	18 (12.6)	1 (0.7)	143
Yes	9 (33.3)	12 (44.4)	2 (7.4)	4 (14.8)	27
Total	114	31	20	5	170

Other G1-G4 toxicities observed were: gastrointestinal in 14.7% of patients, dermatological in 3.6%, liver in 6.6%, pulmonary in 6.5%. Interestingly, 19.4% of patients reported having pain, especially osteo-muscular, abdominal and at tumor site. Eleven SAEs were reported by 10 patients and only two were deemed to be related to treatment (one febrile neutropenia and jaundice and one hypoesthesia and weakness of the lower limb with fever).

### Evaluation of the quality of life

QoL questionnaires were available for 74 patients (75.5%) at the 3^rd^ cycle of eribulin, out of 98 patients with a QoL evaluation at baseline. No significant differences in global health status as well as in physical, social, emotional, cognitive and role functioning were observed between baseline and 3^rd^ cycle questionnaires (Additional file 1: Table S3). Among the 9 reported items/symptoms, a statistically significant worsening was observed only for fatigue (difference =  + 5.4, p = 0.021) and nausea/vomiting (difference + 4.4, p = 0.032). Regarding the QLQ-BR23, a statistically significant worsening was observed in body image perception (difference = -5.9, p = 0.019) and in some side effects of the treatment (difference =  + 6.2, p < 0.001) among 7 items.

As expected, a statistically significant decrease of physical, role functioning, and a worsening in global health status was observed at the end of treatment (Additional file 1: Table S4) and was associated with a worsening of symptoms (fatigue, dyspnea and constipation).

### Genotyping results

Fifteen SNPs were selected based on their reported association with the neurotoxicity of several anticancer agents [14, 16, 19–25]. Additional file 1: Table S5 reports the frequency of variants of all the 15 SNPs and the prevalence of each polymorphism was compared with the expected prevalence in Europe. In most cases, concordance was found between the observed prevalence and the expected one.

Among the 15 SNPs, a statistically significant association with neuropathy was detected for rs2233335 (in *NDRG1* gene; p-value fisher test < 0.001) and rs7214723 (in *CAMKK1* gene; p-value fisher test = 0.034). Regarding the polymorphisms rs7001034 (in *FZD3* gene) and rs242557 (in *MAPT* gene) a higher grade of neurotoxicity was observed in the presence of the allele A (p-value X^2^ for trend = 0.012 and 0.044 for RS7001034 and rs242557, respectively).

To better understand the association between SNPs and neurotoxicity, exploring the allele associated to a higher risk of neurotoxicity, the recessive and dominant model were the salt form investigated. The genotype T/T of the polymorphism rs2233335 (T/T vs. G/G-G/T, p < 0.001 for both fisher and X^2^ for trend) and the genotype T/T of the polymorphism rs7214723 (T/T vs. C/C–C/T, p = 0.008 and p = 0.026 for fisher and X^2^ for trend, respectively) were associated with a higher grade of neurotoxicity (p < 0.001). Considering the neurotoxicity as severe (grade > 1) and non-severe (grade 0–1) the genotype T/T of the polymorphism rs2233335 (OR_T/T vs. G/G-G/T_ 2.44, 95%CI 1.01—5.89, p = 0.047) and the genotype T/T of the polymorphism rs7214723 (OR_T/T vs. G/G-G/T_ 2.56, 95%CI 1.05 – 6.27, p = 0.039) confirmed their association with a higher risk of neurotoxicity (Table 4). The allelic variant G of the polymorphism rs242557 was associated with a statically significant decrease in the occurrence of severe neurotoxicity (OR_G/G vs. A/G vs. A/A_ 0.47, 95%CI 0.23—0.97, p = 0.042). For rs7001034 in the *FZD3* gene, no statistically significant association was detected (OR_G/G vs. A/G vs. A/A_ 0.60, 95%CI 0.32 – 1.15, p = 0.122).

**Table 4:** Relationship using dominant or recessive model between polymorphism and neuropathy

	*G0 N* = *105*	*G1 N* = *30*	*G2 N* = *19*	*G3 N* = *5*	*Fisher exact test (F) test for trend (T) P-value*
*rs2233335—NDRG1—model1—n (%)*					
G/G or G/T	79 (77.5)	12 (11.8)	7 (6.9)	4 (3.9)	F: < 0.001
T/T	26 (45.6)	18 (31.6)	12 (21.1)	1 (1.8)	T: < 0.001
*rs2233335—NDRG1—model2—n (%)*					
G/G	25 (78.1)	2 (6.3)	3 (9.4)	2 (6.3)	F: 0.088
T/T or G/T	80 (63.0)	28 (22.0)	16 (12.6)	3 (2.4)	T: 0.210
*rs7214723—CAMKK1—model1—n (%)*					
C/C or C/T	80 (70.8)	20 (17.7)	8 (7.1)	5 (4.4)	F: 0.008
T/T	23 (52.3)	10 (22.7)	11 (25.0)	0 (0.0)	T: 0.026
Missing	2	0	0	0	
*rs7214723—CAMKK1—model2—n (%)*					
C/C	32 (68.1)	8 (17.0)	5 (10.6)	2 (4.3)	F: 0.892
T/T or C/T	71 (64.5)	22 (20.0)	14 (12.7)	3 (2.7)	T: 0.729
Missing	2	0	0	0	

N: Number of subjects. **G**: Grade.

## Discussion

The treatment of MBC is a challenge in oncology and for several years no advances in overall survival have been observed [26]. New targeted therapies which have become available are changing the natural history of MBC, and new goals have been achieved [3, 27, 28]. Among the different types of chemotherapy, which remain a backbone in this setting, eribulin has demonstrated an improvement in OS in patients with Her2-negative MBC after treatment with anthracyclines/taxanes [10]. As stated in the International Consensus Conference on advanced breast cancer, preserving QoL and avoiding treatment related adverse events are important issues for patients with MBC, since it is a chronic and lethal disease [2]. Eribulin represents a therapeutic option for MBC and as its clinical use will increase with time, a better knowledge of its safety profile outside of clinical trials is warranted. Several retrospective studies have been published on the safety of eribulin in a real life setting [29–31]; however, few prospective studies, aimed at thoroughly evaluating its safety and efficacy in daily clinical practice are available [32, 33]. The PAINTER study was designed to investigate the tolerability of eribulin in a real life setting and to study the association between the onset of neurotoxicity and specific gene polymorphisms. The trial enrolled pretreated MBC patients with characteristics typical of this population: a mean age of 59 years old, 5 median lines of previous chemotherapy for the metastatic setting, up to 18 cycles and mainly Luminal B Her2-negative disease (65%). In the PAINTER study, women received a median of 4,5 cycles of eribulin (range 1–23), and this data are slightly higher than those reported in the EMBRACE and in the TROTTER studies, where patients received 4 lines (range 1–7) and 3 lines (1–10), respectively.

Regarding efficacy, the median OS was 12 months (Q1-Q3: 6.4–21.7 months), in some ways similar to other studies: in EMBRACE the median OS was 13.1 months (95% CI 11.8–14.3) and in the pooled analysis of the 2 phase III studies, the median OS in the ITT population was 15.2 months [12]. The Painter OS is in line with what was observed in real life studies: an OS of 10.1 months (95% CI: 8.1–13), 11.6 months (0.6–33.3 months; 95% CI 8.7–14.5) and 13.53 months (95% CI9.39–17.67 months) [29–31]. Interestingly in the VESPRY study the median OS was 31.8 months (CI 95% 27.9–34.4) and as the authors reported, compared with both these pivotal trials [29–31], there was almost a 2.5 fold increase in OS.This clinical benefit can be partially explained by the fact that the patients who received eribulin were not heavily pretreated, as all patients were in the third line of treatment [34]. Also, Gamucci et al., who reported a median OS of 14.3 months (95%CI, 11.7–16.8) pointed out that a significant improvement in response was observed when eribulin was given as third-line treatment (p = 0.02) [35].

Our study confirms that, even in a heavily pretreated setting, eribulin is well tolerated in fact only 3.5% of patients discontinued treatment because of toxicity /severe adverse events. Most adverse events occur within the 6^th^ cycle; in fact, the risk of interrupting or reducing the dose of eribulin is higher as in the first cycles than in subsequent ones. This is why some patients could continue the treatment for a very long time, up to 23 cycles. Among other G1–G4 toxicities it should be noted that osteo-muscular, abdominal and in tumor site pain was observed in 20% of patients. While this side effect has been described after treatment with other drugs [36], it has never been reported after eribulin treatment.

Most of the enrolled patients had been previously treated with taxanes (97%), a well known neurotoxic drug, similarly to other studies [10, 35]. Interestingly, in our study, 15% of patients reported having neurotoxicity before starting eribulin and this was related to previous treatments; this data are often unreported, but it is very important and must be considered when a patient is evaluated for treatment with eribulin.

Regarding severe toxicity, 38.2% patients experienced at least one severe adverse event; in particular neutropenia in 15.3%of patients, neurotoxicity in 14.7% and asthenia in 5.9%. In our opinion, neuropathy is probably under-reported in other studies [31, 33], while in the PAINTER study the incidence of neurotoxicity was accurately reported thanks to the way the information was collected. In fact, at each cycle a specific question was asked regarding the most frequent toxicities including neurotoxicity. Another possible explanation involves the characteristics of the study population, such as the starting dose of eribulin, previous radiotherapy, and hemoglobin levels at baseline, as identified by Tsurutani et al. as significantly associated with peripheral neuropathy [37]. Anyway, it is clear that eribulin causes peripheral neuropathy; Zhao et al. demonstrated that eribulin-treated subjects (both with breast cancer and liposarcoma) had a significantly increased risk of all-grade (RR, 2.00; 95% CI, 1.70–2.35; p = 0.008) and high-grade (RR, 3.68; 95% CI, 2.30–5.89; p < 0.001) neurotoxicity [38].

A part of the PAINTER study was dedicated to exploring the role of polymorphisms and their relationship with neuropathy. Fifteen SNPs were analyzed and 2 polymorphisms, rs2233335 (T/T) in the NDRG1 gene and rs7214723 (T/T) in the CAMKK1 gene, were associated with eribulin-induced severe neurotoxicity. Regarding rs7001034 in the *FZD3* gene and rs242557 in the *MAPT* gene, an association with neurotoxicity was observed, in particular allele A increased the occurrence of ineuropathy, although it was not confirmed for severe neuropathy. The functions of the involved genes are different; NDRG1 (N-myc downstream regulated gene-1) is a stress response protein involved in multiple cellular pathways, including the endoplasmic reticulum stress response; CAMKK1 (calcium/calmodulin-dependent protein kinase kinase-1) is a serine/threonine kinase that is activated by an increase in intracellular Ca2 + levels and Ca2 + /calmodulin binding; FZD3 is a member of the frizzled gene family, which has been shown to play a role in neurite outgrowth and nerve development; finally, MAPT (microtubule associated protein Tau) is involved in tubulin assembly and polymerization [39–42]. The different mechanisms that underlie the onset of neuropathy are unknown and many studies are exploring them, even in new drugs, like nab-paclitaxel compared to paclitaxel. This suggests that the selection of the nab-paclitaxel regimen should be individualized based on the clinical context and potentially supported by pharmacogenetic analysis [43].

After three cycles of eribulin, QoL questionnaires showed that the global health status was overall preserved, including social, physical and emotional roles. Patients reported a worsening of fatigue and nausea induced by treatment, while no differences were found in the other 7 symptoms and items investigated. In the metastatic setting, the preservation of a good quality of life is essential and our data show that treatment with eribulin allows patients to maintain good health. The global worsening of health status and symptoms at the end of treatment evaluation, was indeed observed at disease progression.

### Limits of the study

Few papers have been published on the efficacy and tolerability of eribulin in a real-life setting [44] and only a small part of them were prospective in nature, suggesting that the data that we reported, give a clearer picture of the tolerability of treatment with eribulin. There are, however, some limitations. First of all, data for genetic analysis set were collected only for 159/180 patients so polymorphism results were not available for all patients. Moreover, the overall population was composed of patients with heterogeneous subtypes (as expected in the real-word setting of the study), and this limited the possibility of specific toxicity analysis in biological subtypes. For the same reason there was a huge heterogeneity in previous treatments.

Regarding genotype analysis, even if the study was multicentric, it was conducted in a specific geographic area (Italy), which could have some implications when the association of specific SNPs and toxicity are considered. Indeed, while it has recently been reported that similar patterns of distribution for the majority of the 65 variant alleles considered exist among super-populations (African, Admixed Americans, East Asian, European and South Asian) [45], we cannot exclude that some heterogeneity might exist in the SNPs considered in our study.

## Conclusions

The PAINTER study offers a wide spectrum of information on the tolerability of eribulin. In fact, the prospective nature of the study allowed the investigators to collect many items based on previous studies. As expected, fatigue, peripheral neuropathy and neutropenia were the most common toxicities, but few patients experienced severe AEs. Toxicities rarely led to drug discontinuation, even if schedule and dosage modifications were common, as can be frequently observed in heavily pretreated patients. Patients reported outcomes show the preservation of a satisfactory global health status during the first three cycles of eribulin and a progressive worsening of symptoms at the end of treatment. To the best of our knowledge, this is the first study that demonstrates a correlation between SNPs and neuropathy in patients treated with eribulin. Ongoing studies are trying to understand the molecular mechanisms at the basis of this association. These data, if confirmed by other studies, will allow a tailored treatment with eribulin, addressing the proper use of the drug while avoiding useless toxicity.

## Supplementary information

**Additional file 1. Table S1. **List of the SNPs analysed.** Table S2. **Other uncommon Adverse Events–maximum grade.** Table S3. **QOL scores – change from baseline to third cycle.** Table S4. **QOL scores – change from baseline to end of treatment.** Table S5. **Frequency of variants of all the 15 SNPs and the prevalence of each polymorphism was compared with the expected prevalence in Europe.** Table S6. **Number of eribulin cycles based on previous neurotoxicity.

## Data Availability

The datasets used and/or analyzed during the current study are available from the corresponding author on reasonable request.

## Abbreviations

AEs: Adverse events
SAEs: Severe AE
DOT: Duration of treatment
G: Grade
MBC: Metastatic breast cancer
OR: Odds ratio
OS: Overall survival
PN: Peripheral neuropathy
pts: Patients
QoL: Quality of life
SNPs: Single Nucleotide Polymorphisms
TPC: Treatment of physician’s choice
