# Supplementary material for: Tolerability of Eribulin and correlation between polymorphisms and neuropathy in an unselected population of female patients with metastatic breast cancer: results of the multicenter, single arm, phase IV PAINTER study
Source: Breast Cancer Res. 2022 Oct 28;24:71. doi: 10.1186/s13058-022-01560-w (PMC9615373; doi:10.1186/s13058-022-01560-w)
Supplement: Supplementary file 1 — Additional file 1. Table S1. List of the SNPs analysed. Table S2. Other uncommon Adverse Events–maximum grade. Table S3. QOL scores – change from baseline to third cycle. Table S4. QOL scores – change from baseline to end of treatment. Table S5. Frequency of variants of all the 15 SNPs and the prevalence of each polymorphism was compared with the expected prevalence in Europe. Table S6. Number of eribulin cycles based on previous neurotoxicity. [file 13058_2022_1560_MOESM1_ESM.docx]

| **Supplementary Table 1.** List of the SNPs analysed | | | |
| --- | --- | --- | --- |
| **Gene** | **SNP ID** | **Polymorphism** | **MAF** |
| **ABCB1** | rs3213619 | **A**/T | 0.419 |
| **ADRB2** | rs2082382 | A/**G** | 0.184 |
| **CAMKK1** | rs7214723 | C/**T** | 0.279 |
| **Chr.1 68392796 on GRCh38** | rs3125923 | C/**T** |  |
| **BCL6 BCL6** | rs1903216 | C/**T** | 0.48 |
| **EPHA5** | rs7349683 | C/**T** | 0.36 |
| **FANCD2** | rs6442150 | **C**/T |  |
| **FANCD2** | rs6786638 | **C**/G | 0.182 |
| **FGD4** | rs10771973 | **A**/G | 0.31 |
| **FZD3** | rs7001034 | **A**/G | 0.398 |
| **GSTP1** | rs1138272 | C/**T** | 0.036 |
| **MAPT** | rs242557 | **A**/G/T |  |
| **mir3194** | rs228832 | A/**G** |  |
| **NDRG1** | rs2233335 | A/**C** | 0.38 |
| **XKR4** | rs4737264 | A/**C** | 0.22 |
| **SNP**: single nucleotide polymorphism; **MAF**: minor allele frequency | | | |

| **Supplementary Table 2.** Other uncommon Adverse Events– maximum grade | | | | | | | |
| --- | --- | --- | --- | --- | --- | --- | --- |
| **Toxicity – N=170** | **G0 n (%)** | **G1 n (%)** | **G2 n (%)** | **G3 n (%)** | **G4 n (%)** | **G5 n (%)** | **Severe toxicity**  **(G3+G4+G5) n (%) [%95%CI]** |
| **Blood/bone marrow** | 153 (90.0) | 5 (2.9) | 8 (4.7) | 3 (1.8) | 1 (0.6) | 0 (0.0) | 4 (2.4) [0.65 - 5.91] |
| - Hemoglobin (Hgb) | 158 (92.9) | 4 (2.4) | 7 (4.1) | 1 (0.6) | 0 (0.0) | 0 (0.0) | 1 (0.6) |
| - Leukocytes (total) | 167 (98.2) | 1 (0.6) | 2 (1.2) | 0 (0.0) | 0 (0.0) | 0 (0.0) | 0 (0.0) |
| - Lymphopenia | 169 (99.4) | 1 (0.6) | 0 (0.0) | 0 (0.0) | 0 (0.0) | 0 (0.0) | 0 (0.0) |
| - Neutrophils | 165 (97.1) | 1 (0.6) | 1 (0.6) | 2 (1.2) | 1 (0.6) | 0 (0.0) | 3 (1.8) |
| - Platelets | 167 (98.2) | 3 (1.8) | 0 (0.0) | 0 (0.0) | 0 (0.0) | 0 (0.0) | 0 (0.0) |
| - Transfusion: pRBCs | 169 (99.4) | 0 (0.0) | 0 (0.0) | 1 (0.6) | 0 (0.0) | 0 (0.0) | 1 (0.6) |
| **Cardiovascular (general)** | 167 (98.2) | 1 (0.6) | 1 (0.6) | 1 (0.6) | 0 (0.0) | 0 (0.0) | 1 (0.6) [0.02 - 3.23] |
| - Edema | 168 (98.8) | 0 (0.0) | 1 (0.6) | 1 (0.6) | 0 (0.0) | 0 (0.0) | 1 (0.6) |
| - Hypertension | 169 (99.4) | 1 (0.6) | 0 (0.0) | 0 (0.0) | 0 (0.0) | 0 (0.0) | 0 (0.0) |
| **Coagulation** | 169 (99.4) | 0 (0.0) | 1 (0.6) | 0 (0.0) | 0 (0.0) | 0 (0.0) | 0 (0.0) |
| - Fatigue (asthenia) | 169 (99.4) | 0 (0.0) | 1 (0.6) | 0 (0.0) | 0 (0.0) | 0 (0.0) | 0 (0.0) |
| **Dermatology/skin** | 164 (96.5) | 3 (1.8) | 3 (1.8) | 0 (0.0) | 0 (0.0) | 0 (0.0) | 0 (0.0) |
| - Alopecia | 169 (99.4) | 1 (0.6) | 0 (0.0) | 0 (0.0) | 0 (0.0) | 0 (0.0) | 0 (0.0) |
| - Erythema multiforme | 169 (99.4) | 1 (0.6) | 0 (0.0) | 0 (0.0) | 0 (0.0) | 0 (0.0) | 0 (0.0) |
| - Pruritus | 169 (99.4) | 0 (0.0) | 1 (0.6) | 0 (0.0) | 0 (0.0) | 0 (0.0) | 0 (0.0) |
| - Rash/desquamation | 169 (99.4) | 1 (0.6) | 0 (0.0) | 0 (0.0) | 0 (0.0) | 0 (0.0) | 0 (0.0) |
| - Dermatology/Skin-Other (Specify in NOTE) | 168 (98.8) | 0 (0.0) | 2 (1.2) | 0 (0.0) | 0 (0.0) | 0 (0.0) | 0 (0.0) |
| **Gastrointestinal** | 145 (85.3) | 17 (10.0) | 5 (2.9) | 3 (1.8) | 0 (0.0) | 0 (0.0) | 3 (1.8) [0.37 - 5.07] |
| - Anorexia | 169 (99.4) | 0 (0.0) | 1 (0.6) | 0 (0.0) | 0 (0.0) | 0 (0.0) | 0 (0.0) |
| - Diarrhea | 169 (99.4) | 1 (0.6) | 0 (0.0) | 0 (0.0) | 0 (0.0) | 0 (0.0) | 0 (0.0) |
| - Dyspepsia/heartburn | 167 (98.2) | 2 (1.2) | 1 (0.6) | 0 (0.0) | 0 (0.0) | 0 (0.0) | 0 (0.0) |
| - Dysphagia | 168 (98.8) | 1 (0.6) | 1 (0.6) | 0 (0.0) | 0 (0.0) | 0 (0.0) | 0 (0.0) |
| - Gastritis | 169 (99.4) | 1 (0.6) | 0 (0.0) | 0 (0.0) | 0 (0.0) | 0 (0.0) | 0 (0.0) |
| - Mouth dryness | 169 (99.4) | 1 (0.6) | 0 (0.0) | 0 (0.0) | 0 (0.0) | 0 (0.0) | 0 (0.0) |
| - Mucositis | 163 (95.9) | 5 (2.9) | 2 (1.2) | 0 (0.0) | 0 (0.0) | 0 (0.0) | 0 (0.0) |
| - Vomiting | 162 (95.3) | 5 (2.9) | 1 (0.6) | 2 (1.2) | 0 (0.0) | 0 (0.0) | 2 (1.2) |
| - Gastrointestinal-Other (Specify in NOTE) | 163 (95.9) | 6 (3.5) | 0 (0.0) | 1 (0.6) | 0 (0.0) | 0 (0.0) | 1 (0.6) |
| **Hemorrhage** | 169 (99.4) | 1 (0.6) | 0 (0.0) | 0 (0.0) | 0 (0.0) | 0 (0.0) | 0 (0.0) |
| - Hemorrhage-Other  (Specify site in NOTE) | 169 (99.4) | 1 (0.6) | 0 (0.0) | 0 (0.0) | 0 (0.0) | 0 (0.0) | 0 (0.0) |
| Hepatic | 159 (93.5) | 3 (1.8) | 3 (1.8) | 4 (2.4) | 1 (0.6) | 0 (0.0) | 5 (2.9) [0.96 - 6.73] |
| - Alkaline phosphatase | 169 (99.4) | 0 (0.0) | 1 (0.6) | 0 (0.0) | 0 (0.0) | 0 (0.0) | 0 (0.0) |
| - Bilirubin | 169 (99.4) | 0 (0.0) | 1 (0.6) | 0 (0.0) | 0 (0.0) | 0 (0.0) | 0 (0.0) |
| - GGT | 168 (98.8) | 0 (0.0) | 0 (0.0) | 1 (0.6) | 1 (0.6) | 0 (0.0) | 2 (1.2) |
| - SGOT (AST) | 161 (94.7) | 6 (3.5) | 2 (1.2) | 1 (0.6) | 0 (0.0) | 0 (0.0) | 1 (0.6) |
| - SGPT (ALT) Hepatic-Other (Specify in NOTE) | 162 (95.3) | 5 (2.9) | 1 (0.6) | 2 (1.2) | 0 (0.0) | 0 (0.0) | 2 (1.2) |
| **Infection/febrile neutropenia** | 162 (95.3) | 2 (1.2) | 1 (0.6) | 2 (1.2) | 3 (1.8) | 0 (0.0) | 5 (2.9) [0.96 - 6.73] |
| - Catheter-related infection | 169 (99.4) | 1 (0.6) | 0 (0.0) | 0 (0.0) | 0 (0.0) | 0 (0.0) | 0 (0.0) |
| - Febrile neutropenia | 168 (98.8) | 0 (0.0) | 0 (0.0) | 1 (0.6) | 1 (0.6) | 0 (0.0) | 2 (1.2) |
| - Infection with grade 3 or 4 neutropenia | 168 (98.8) | 0 (0.0) | 1 (0.6) | 0 (0.0) | 1 (0.6) | 0 (0.0) | 1 (0.6) |
| - Infection without neutropenia | 167 (98.2) | 1 (0.6) | 2 (1.2) | 0 (0.0) | 0 (0.0) | 0 (0.0) | 0 (0.0) |
| - Infection/Febrile Neutropenia-Other  (Specify in NOTE) | 168 (98.8) | 0 (0.0) | 0 (0.0) | 1 (0.6) | 1 (0.6) | 0 (0.0) | 2 (1.2) |
| **Lymphatics** | 168 (98.8) | 2 (1.2) | 0 (0.0) | 0 (0.0) | 0 (0.0) | 0 (0.0) | 0 (0.0) |
| - Lymphedema | 169 (99.4) | 1 (0.6) | 0 (0.0) | 0 (0.0) | 0 (0.0) | 0 (0.0) | 0 (0.0) |
| - Lymphatics-Other  (Specify in NOTE) | 169 (99.4) | 1 (0.6) | 0 (0.0) | 0 (0.0) | 0 (0.0) | 0 (0.0) | 0 (0.0) |
| **Metabolic/laboratory** | 163 (95.9) | 2 (1.2) | 3 (1.8) | 2 (1.2) | 0 (0.0) | 0 (0.0) | 2 (1.2) [0.14 - 4.19] |
| - Metabolic/Laboratory-Other (Specify in NOTE) | 163 (95.9) | 2 (1.2) | 3 (1.8) | 2 (1.2) | 0 (0.0) | 0 (0.0) | 2 (1.2) |
| **Musculoskeletal** | 166 (97.6) | 3 (1.8) | 1 (0.6) | 0 (0.0) | 0 (0.0) | 0 (0.0) | 0 (0.0) |
| - Arthritis | 169 (99.4) | 1 (0.6) | 0 (0.0) | 0 (0.0) | 0 (0.0) | 0 (0.0) | 0 (0.0) |
| - Musculoskeletal-Other (Specify in NOTE) | 167 (98.2) | 2 (1.2) | 1 (0.6) | 0 (0.0) | 0 (0.0) | 0 (0.0) | 0 (0.0) |
| **Neurology** | 163 (95.9) | 4 (2.4) | 3 (1.8) | 0 (0.0) | 0 (0.0) | 0 (0.0) | 0 (0.0) |
| - Confusion | 167 (98.2) | 2 (1.2) | 1 (0.6) | 0 (0.0) | 0 (0.0) | 0 (0.0) | 0 (0.0) |
| - Depressed level of consciousness | 169 (99.4) | 1 (0.6) | 0 (0.0) | 0 (0.0) | 0 (0.0) | 0 (0.0) | 0 (0.0) |
| - Dizziness/lightheadedness | 169 (99.4) | 1 (0.6) | 0 (0.0) | 0 (0.0) | 0 (0.0) | 0 (0.0) | 0 (0.0) |
| - Insomnia | 169 (99.4) | 0 (0.0) | 1 (0.6) | 0 (0.0) | 0 (0.0) | 0 (0.0) | 0 (0.0) |
| - Memory loss | 169 (99.4) | 1 (0.6) | 0 (0.0) | 0 (0.0) | 0 (0.0) | 0 (0.0) | 0 (0.0) |
| - Speech impairment  (e.g., dysphasia or aphasia) | 169 (99.4) | 0 (0.0) | 1 (0.6) | 0 (0.0) | 0 (0.0) | 0 (0.0) | 0 (0.0) |
| - Vertigo | 169 (99.4) | 1 (0.6) | 0 (0.0) | 0 (0.0) | 0 (0.0) | 0 (0.0) | 0 (0.0) |
| - Neurology-Other  (Specify in NOTE) | 168 (98.8) | 2 (1.2) | 0 (0.0) | 0 (0.0) | 0 (0.0) | 0 (0.0) | 0 (0.0) |
| **Ocular/visual** | 168 (98.8) | 2 (1.2) | 0 (0.0) | 0 (0.0) | 0 (0.0) | 0 (0.0) | 0 (0.0) |
| - Conjunctivitis | 168 (98.8) | 2 (1.2) | 0 (0.0) | 0 (0.0) | 0 (0.0) | 0 (0.0) | 0 (0.0) |
| **Pain** | 137 (80.6) | 22 (12.9) | 7 (4.1) | 4 (2.4) | 0 (0.0) | 0 (0.0) | 4 (2.4) [0.65 - 5.91] |
| - Abdominal pain or cramping | 163 (95.9) | 7 (4.1) | 0 (0.0) | 0 (0.0) | 0 (0.0) | 0 (0.0) | 0 (0.0) |
| - Arthralgia (joint pain) | 169 (99.4) | 1 (0.6) | 0 (0.0) | 0 (0.0) | 0 (0.0) | 0 (0.0) | 0 (0.0) |
| - Bone pain | 161 (94.7) | 2 (1.2) | 5 (2.9) | 2 (1.2) | 0 (0.0) | 0 (0.0) | 2 (1.2) |
| - Chest pain  (non-cardiac and non-pleuritic) | 168 (98.8) | 1 (0.6) | 0 (0.0) | 1 (0.6) | 0 (0.0) | 0 (0.0) | 1 (0.6) |
| - Headache | 165 (97.1) | 4 (2.4) | 0 (0.0) | 1 (0.6) | 0 (0.0) | 0 (0.0) | 1 (0.6) |
| - Hepatic pain | 169 (99.4) | 0 (0.0) | 1 (0.6) | 0 (0.0) | 0 (0.0) | 0 (0.0) | 0 (0.0) |
| - Myalgia (muscle pain) | 164 (96.5) | 5 (2.9) | 1 (0.6) | 0 (0.0) | 0 (0.0) | 0 (0.0) | 0 (0.0) |
| - Tumor pain  (onset or exacerbation of tumor pain due to treatment) | 168 (98.8) | 1 (0.6) | 1 (0.6) | 0 (0.0) | 0 (0.0) | 0 (0.0) | 0 (0.0) |
| - Pain-Other  (Specify in NOTE) | 163 (95.9) | 7 (4.1) | 0 (0.0) | 0 (0.0) | 0 (0.0) | 0 (0.0) | 0 (0.0) |
| **Pulmonary** | 159 (93.5) | 5 (2.9) | 3 (1.8) | 2 (1.2) | 0 (0.0) | 1 (0.6) | 3 (1.8) [0.37 - 5.07] |
| - Cough | 165 (97.1) | 1 (0.6) | 4 (2.4) | 0 (0.0) | 0 (0.0) | 0 (0.0) | 0 (0.0) |
| - Dyspnea (shortness of breath) | 164 (96.5) | 4 (2.4) | 0 (0.0) | 1 (0.6) | 0 (0.0) | 1 (0.6) | 2 (1.2) |
| - Pleural effusion  (non-malignant) | 169 (99.4) | 0 (0.0) | 0 (0.0) | 1 (0.6) | 0 (0.0) | 0 (0.0) | 1 (0.6) |
| - Pneumothorax | 169 (99.4) | 1 (0.6) | 0 (0.0) | 0 (0.0) | 0 (0.0) | 0 (0.0) | 0 (0.0) |
| - Pulmonary-Other  (Specify in NOTE) | 169 (99.4) | 1 (0.6) | 0 (0.0) | 0 (0.0) | 0 (0.0) | 0 (0.0) | 0 (0.0) |
| **Renal/genitourinary** | 169 (99.4) | 1 (0.6) | 0 (0.0) | 0 (0.0) | 0 (0.0) | 0 (0.0) | 0 (0.0) |
| - Incontinence | 169 (99.4) | 1 (0.6) | 0 (0.0) | 0 (0.0) | 0 (0.0) | 0 (0.0) | 0 (0.0) |
| **Syndromes (not included in previous categories)** | 158 (92.9) | 5 (2.9) | 6 (3.5) | 1 (0.6) | 0 (0.0) | 0 (0.0) | 1 (0.6) [0.02 - 3.23] |
| - Syndromes-Other  (Specify in NOTE) | 158 (92.9) | 5 (2.9) | 6 (3.5) | 1 (0.6) | 0 (0.0) | 0 (0.0) | 1 (0.6) |
| **Legend: N**: Number of subjects; **G**: Grade. **pRBc**: packed red blood cells. | | | | | | | |

| **Supplementary Table 3.** QOL scores – change from baseline to third cycle |
| --- |

|  | **Baseline (T0)**  Mean (SD) **N=74** | **Third cycle**  Mean (SD) **N=74** | **Difference  3^rd^cycle-T0 N=74** | **P-value** |
| --- | --- | --- | --- | --- |
| **EORTC QLQ-C30** |  |  |  |  |
| ***Functional scales*** |  |  |  |  |
| Physical Functioning | 74.7 (18.7) | 73.8 (19.2) | -0.9 (15.4) | 0.616 |
| Role Functioning | 73.4 (27.6) | 73.0 (25.5) | -0.5 (20.3) | 0.849 |
| Emotional Functioning | 72.4 (20.2) | 73.2 (20.0) | 0.8 (17.0) | 0.691 |
| Cognitive Functioning | 88.5 (19.7) | 85.8 (19.4) | -2.7 (17.2) | 0.181 |
| Social Functioning | 79.3 (26.1) | 78.2 (26.3) | -1.1 (23.9) | 0.689 |
| **Global health status / QoL** | 55.9 (20.0) | 54.0 (20.0) | -1.9 (17.9) | 0.365 |
| ***Symptom scales / item*** |  |  |  |  |
| Fatigue | 33.2 (24.9) | 38.6 (25.3) | 5.4 (19.7) | 0.021 |
| Nausea / Vomiting | 5.8 (13.3) | 10.2 (19.4) | 4.4 (17.6) | 0.032 |
| Pain | 25.0 (27.9) | 25.2 (27.2) | 0.2 (19.4) | 0.921 |
| Dyspnea | 18.2 (25.9) | 19.6 (25.2) | 1.3 (22.9) | 0.615 |
| Insomnia | 24.4 (30.2) | 23.6 (24.4) | -0.9 (24.5) | 0.754 |
| Appetite loss | 13.5 (23.4) | 15.8 (24.8) | 2.3 (21.6) | 0.373 |
| Constipation | 10.8 (23.5) | 11.3 (23.6) | 0.5 (26.7) | 0.885 |
| Diarrhea | 7.2 (16.8) | 5.4 (14.6) | -1.8 (21.3) | 0.469 |
| Financial Problems | 14.0 (27.0) | 11.3 (25.4) | -2.7 (14.3) | 0.109 |
| **QLQ-BR23** |  |  |  |  |
| ***Functional scales*** |  |  |  |  |
| Body image | 77.8 (30.3) | 71.9 (31.1) | -5.9 (20.7) | 0.019 |
| Sexual functioning | 6.7 (14.7) | 5.6 (13.7) | -1.2 (13.5) | 0.470 |
| Future perspective | 54.0 (31.6) | 57.3 (34.4) | 3.3 (32.4) | 0.396 |
| ***Symptom scales / items*** |  |  |  |  |
| Systematic therapy side effects | 13.8 (15.0) | 20.0 (17.1) | 6.2 (13.8) | <0.001 |
| Breast symptoms | 8.5 (13.0) | 8.1 (11.6) | -0.4 (11.3) | 0.794 |
| Arm symptoms | 14.4 (18.8) | 13.0 (19.1) | -1.4 (11.1) | 0.289 |
| Upset by hair loss | 33.3 (33.3) | 33.3 (33.3) | 0.0 (0.0) | . |
| **Legend:** SD: standard deviation. A high score for a functional scale represents a high / healthy level of functioning, a high score for the global health status / QoL represents a high QoL, but a high score for a symptom scale / item represents a high level of symptomatology / problems. | | | | |

| **Supplementary Table 4. QOL scores – change from baseline to end of treatment** |
| --- |

|  | **Baseline (T0)**  Mean (SD) **N=98** | **End of treatment (EoT)**  Mean (SD) **N=98** | **Difference  EoT-T0 N=98** | **P-value** |
| --- | --- | --- | --- | --- |
| **EORTC QLQ-C30** |  |  |  |  |
| ***Functional scales*** | | | | |
| Physical Functioning | 74.1 (18.8) | 69.2 (22.1) | -4.9 (16.1) | 0.003* |
| Role Functioning | 72.4 (27.8) | 66.0 (27.7) | -6.5 (20.8) | 0.003* |
| Emotional Functioning | 72.0 (21.6) | 71.1 (21.3) | -0.9 (18.6) | 0.619 |
| Cognitive Functioning | 86.2 (20.5) | 82.5 (22.8) | -3.7 (20.1) | 0.068 |
| Social Functioning | 76.9 (28.0) | 74.8 (27.9) | -2.0 (26.8) | 0.453 |
| **Global health status / QoL** | 55.9 (20.0) | 50.5 (19.9) | -5.4 (20.2) | 0.010* |
| ***Symptom scales / item*** | | | | |
| Fatigue | 34.8 (25.1) | 41.2 (25.5) | 6.3 (21.8) | 0.005* |
| Nausea / Vomiting | 6.1 (12.5) | 6.8 (14.1) | 0.7 (14.3) | 0.640 |
| Pain | 26.4 (27.8) | 29.1 (26.4) | 2.7 (24.0) | 0.265 |
| Dyspnea | 19.7 (26.6) | 25.9 (27.7) | 6.1 (24.1) | 0.014* |
| Insomnia | 26.9 (29.4) | 28.2 (26.8) | 1.4 (27.5) | 0.625 |
| Appetite loss | 15.0 (23.5) | 17.7 (25.0) | 2.7 (25.2) | 0.287 |
| Constipation | 8.9 (21.3) | 14.8 (24.5) | 5.8 (24.1) | 0.019* |
| Diarrhea | 7.8 (17.8) | 6.5 (16.3) | -1.4 (22.4) | 0.549 |
| Financial Problems | 13.3 (26.5) | 11.2 (25.3) | -2.0 (15.7) | 0.202 |
| **QLQ-BR23** |  |  |  |  |
| ***Functional scales*** | | | | |
| Body image | 75.7 (31.3) | 73.7 (30.8) | -2.0 (22.7) | 0.385 |
| Sexual functioning | 7.4 (14.8) | 3.2 (9.9) | -4.3 (14.0) | 0.004* |
| Future perspective | 51.8 (32.3) | 53.9 (34.9) | 2.1 (34.5) | 0.551 |
| ***Symptom scales / items*** | | | | |
| Systematic therapy side effects | 14.7 (14.4) | 21.4 (15.5) | 6.6 (12.7) | <0.001* |
| Breast symptoms | 10.5 (17.5) | 10.7 (17.1) | 0.2 (12.7) | 0.893 |
| Arm symptoms | 16.2 (19.1) | 16.0 (20.9) | -0.2 (13.7) | 0.868 |
| Upset by hair loss | 33.3 (33.3) | 29.6 (30.9) | -3.7 (20.0) | 0.594 |
| **Legend:** SD: standard deviation. A high score for a functional scale represents a high / healthy level of functioning, a high score for the global health status / QoL represents a high QoL, but a high score for a symptom scale / item represents a high level of symptomatology / problems. | | | | |

| **Supplementary Table 5. Frequency of variants of all the 15 SNPs and the prevalence of each polymorphism was compared with the expected prevalence in Europe** | | | |
| --- | --- | --- | --- |
|  | **OVERALL**  **N=159** | **EUROPE**  **Genotype %** | **Chi-squared GOF test**  **P-value** |
| **rs1138272 - GSTP1 - n (%)** | | | |
| C/C | 140 (88.6) | 85.9 | 0.444* |
| C/T | 16 (10.1) | 14.1 |  |
| T/T | 2 (1.3) | 0 |  |
| Missing | 1 |  |  |
| **rs1903216 - close to Bcl6 - n (%)** | | | |
| A/A | 48 (30.4) | 22.3 | 0.204 |
| A/G | 73 (46.2) | 53.3 |  |
| G/G | 37 (23.4) | 24.5 |  |
| Missing | 1 |  |  |
| **rs2082382 ADRB2 - n (%)** | | | |
| A/A | 68 (43.0) | 33.8 | 0.028# |
| A/G | 75 (47.5) | 51.9 |  |
| G/G | 15 (9.5) | 14.3 |  |
| Missing | 1 |  |  |
| **rs7001034 - FZD3 - n (%)** | | | |
| A/A | 19 (12.0) | 15.5 | 0.473 |
| A/G | 76 (48.1) | 46.9 |  |
| G/G | 63 (39.9) | 37.6 |  |
| Missing | 1 |  |  |
| **rs6786638 - FANCD2 - n (%)** | | | |
| C/C | 5 (3.1) | 2.4 | 0.356* |
| C/G | 44 (27.7) | 23.5 |  |
| G/G | 110 (69.2) | 74.2 |  |
| **rs228832 - mir3194 - n (%)** | | | |
| C/C | 92 (57.9) | 59.6 | 0.511 |
| C/T | 54 (34.0) | 34.4 |  |
| T/T | 13 (8.2) | 6 |  |
| **rs10771973 - FGD4 - n (%)** | | | |
| A/A | 25 (16.0) | 10.1 | 0.039# |
| A/G | 60 (38.5) | 43.9 |  |
| G/G | 71 (45.5) | 45.9 |  |
| Missing | 3 |  |  |
| **rs4737264 - XKR4 - n (%)** | | | |
| A/A | 108 (68.8) | 64 | 0.402 |
| A/C | 45 (28.7) | 32.2 |  |
| C/C | 4 (2.5) | 3.8 |  |
| Missing | 2 |  |  |
| **rs3125923 Chr.1 68392796 on GRCh38 - n (%)** | | | |
| A/A | 124 (78.5) | 73.8 | 0.362* |
| A/G | 31 (19.6) | 24.5 |  |
| G/G | 3 (1.9) | 1.8 |  |
| Missing | 1 |  |  |
| **rs7349683 - EPHA5 - n (%)** | | | |
| C/C | 72 (45.3) | 37.4 | 0.118 |
| C/T | 69 (43.4) | 50.3 |  |
| T/T | 18 (11.3) | 12.3 |  |
| **rs2233335 - NDRG1 - n (%)** | | | |
| G/G | 32 (20.1) | 13.1 | 0.029# |
| G/T | 70 (44.0) | 46.1 |  |
| T/T | 57 (35.8) | 40.8 |  |
| **rs7214723 - CAMKK1 - n (%)** | | | |
| C/C | 47 (29.9) | 20.5 | 0.014# |
| C/T | 66 (42.0) | 47.7 |  |
| T/T | 44 (28.0) | 31.8 |  |
| Missing | 2 |  |  |
| **rs3213619 - ABCB1 - n (%)** | | | |
| A/A | 147 (93.0) | 92.4 | 0.834* |
| A/G | 11 (7.0) | 7.4 |  |
| G/G | 0 (0.0) | 0.2 |  |
| Missing | 1 |  |  |
| **rs6442150 - FANCD2 - n (%)** | | | |
| C/C | 5 (3.2) | 2.4 | 0.365* |
| C/T | 43 (27.6) | 23.5 |  |
| T/T | 108 (69.2) | 74.2 |  |
| Missing | 3 |  |  |
| **rs242557 - MAPT - n (%)** | | | |
| A/A | 9 (5.7) | 13.7 | 0.004# |
| A/G | 77 (48.7) | 49.7 |  |
| G/G | 72 (45.6) | 36.6 |  |
| Missing | 1 |  |  |
| **Legend: N**: Number of subjects. **GOF**: Goodness Of Fit. *****: The Chi-square approximation could be inaccurate.  **#**:P-value<0.05 | | | |

**Supplementary Table 6 – Number of eribulin cycles based on previous neurotoxicity**

| Previous Neurotoxicity | N | Mean | SD | Min | Max | Median | Q1 | Q3 |
| --- | --- | --- | --- | --- | --- | --- | --- | --- |
| No | **143** | 5.49 | 3.89 | 1.00 | 22.00 | 4.00 | 3.00 | 7.00 |
| Yes | **27** | 5.93 | 4.19 | 1.00 | 23.00 | 5.00 | 3.00 | 8.00 |
